# Case Report: Intrahepatic cholangiocarcinoma masquerading as a giant hepatic cyst

**DOI:** 10.3389/fonc.2026.1706024

**Published:** 2026-03-24

**Authors:** Zhong Tian, Jianxu Rao, Di Zhang, Jiao Cheng, Xinqi Yang, Yunchen Zhao, Bo Yu, Zhongcong He, Cheng Zhu, Kai Leng, Ni Fu

**Affiliations:** 1 Department of Urology, The Second Affiliated Hospital of Zunyi Medical University, Zunyi, China; 2 Department of Hepatobiliary, The Third Affiliated Hospital of Zunyi Medical University, Zunyi, China; 3 Department of Pathology, The Third Affiliated Hospital of Zunyi Medical University, Zunyi, China; 4 Department of Urology, The Affiliated Hospital of Zunyi Medical University, Zunyi, China

**Keywords:** cholangiocarcinoma, ERCP, giant hepatic cyst, ICC, IPC, IPCC

## Abstract

Even asymptomatic giant hepatic cysts may harbor malignancy. Intraductal papillary carcinoma (IPC) of the liver, a histologic subtype of intrahepatic cholangiocarcinoma (ICC), is relatively uncommon among primary hepatic malignancies. The coexistence of ICC with hepatic cysts is exceedingly rare and often leads to misdiagnosis or missed diagnosis. This case report describes a patient in whom both ICC and a giant hepatic cyst were identified during a routine physical examination. Initial imaging favored a diagnosis of a giant hepatic cyst. Laparoscopic hepatic cystectomy with partial hepatectomy was performed, and histopathologic examination confirmed intraductal papillary carcinoma.

## Introduction

ICC is a malignancy arising from the biliary epithelium of the intrahepatic bile ducts (1). It is the second most common primary liver cancer after hepatocellular carcinoma, accounting for approximately 10–15% of primary hepatic malignancies (2, 3). Hepatic cysts are common benign liver lesions, most typically of congenital origin; acquired forms are less frequent (4, 5). A “giant” hepatic cyst is generally defined as a simple liver cyst larger than 10 cm in diameter. Concomitant ICC and a giant hepatic cyst is exceedingly rare. In this case, routine screening CT identified a giant hepatic cyst, prompting further evaluation. Given cholestatic liver enzyme abnormalities raising concern for biliary obstruction, MRI/MRCP demonstrated common bile duct stones (choledocholithiasis) and a complex cystic-solid mass in the left hepatic lobe, suspicious for a mucinous cystic neoplasm. ERCP with biliary sphincterotomy and stone extraction was performed to relieve obstruction, followed by laparoscopic hepatic cystectomy and partial hepatectomy. Intraoperative frozen-section analysis suggested a tubulovillous adenoma, whereas final histopathology and immunohistochemistry confirmed intraductal papillary adenocarcinoma coexisting with a giant hepatic cyst. Therefore, even large asymptomatic cysts may harbor malignancy, warranting a high index of suspicion to prevent misdiagnosis or missed diagnosis.

## Case report

**Figure 1:**
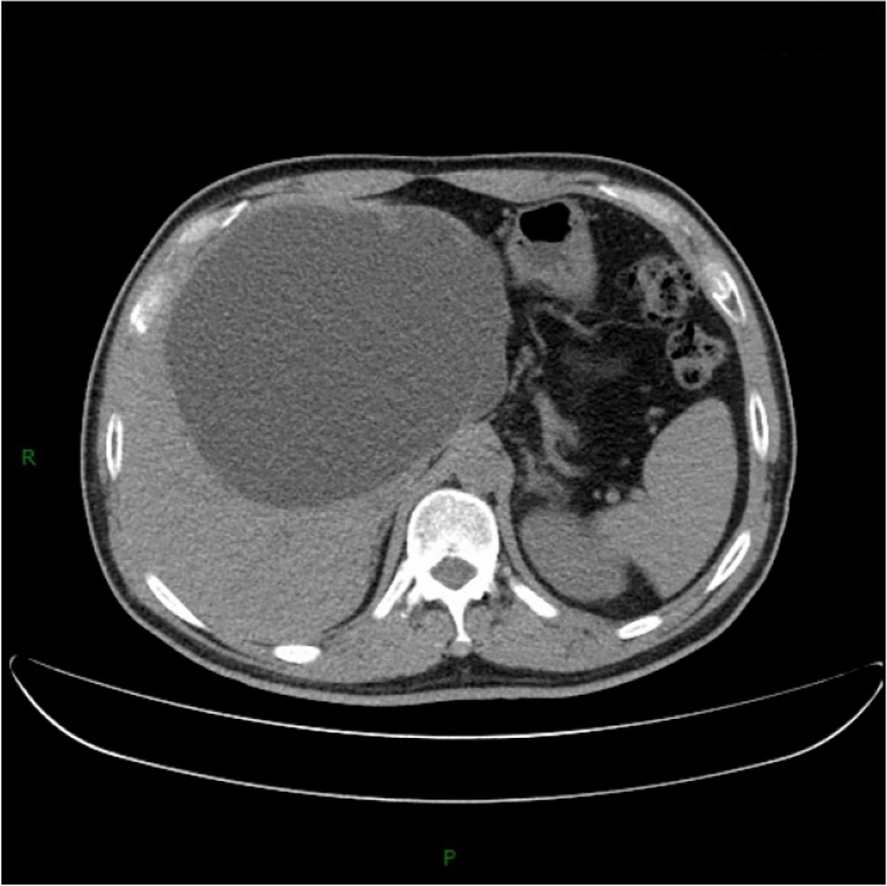
Noncontrast CT of the upper abdomen demonstrates a large cystic lesion in the left hepatic lobe measuring approximately 17 cm in greatest dimension, compatible with a giant hepatic cyst. Further characterization with contrast-enhanced CT of the upper abdomen is recommended.

**Figure 2:**
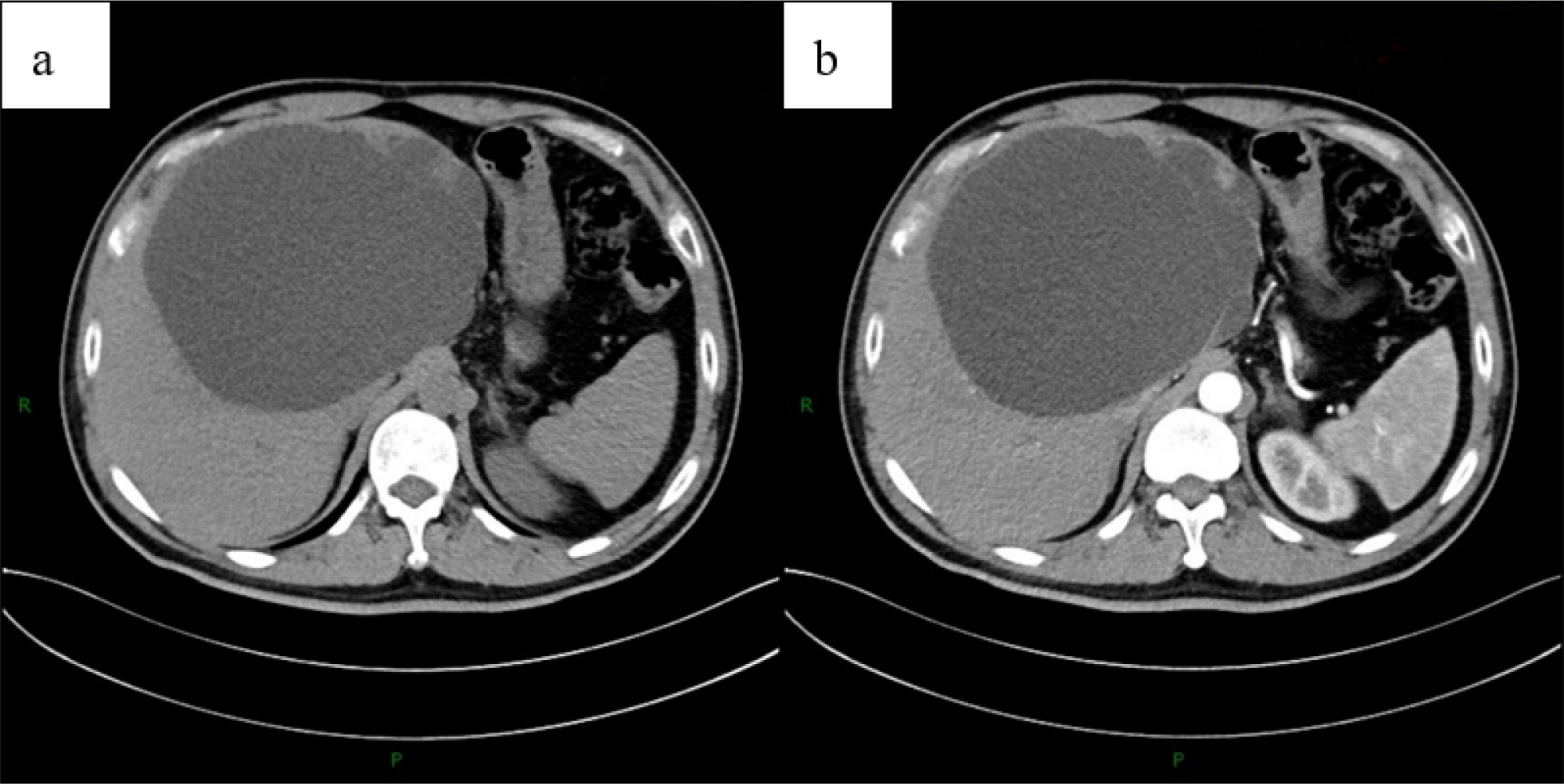
Noncontrast **(a)** and contrast-enhanced **(b)** CT of the upper abdomen demonstrate a round, fluid-attenuation lesion in the left hepatic lobe measuring approximately 16.7 x 13.9 cm, without internal enhancement and causing mass effect on adjacent structures, findings compatible with a large hepatic cyst.

**Figure 3:**
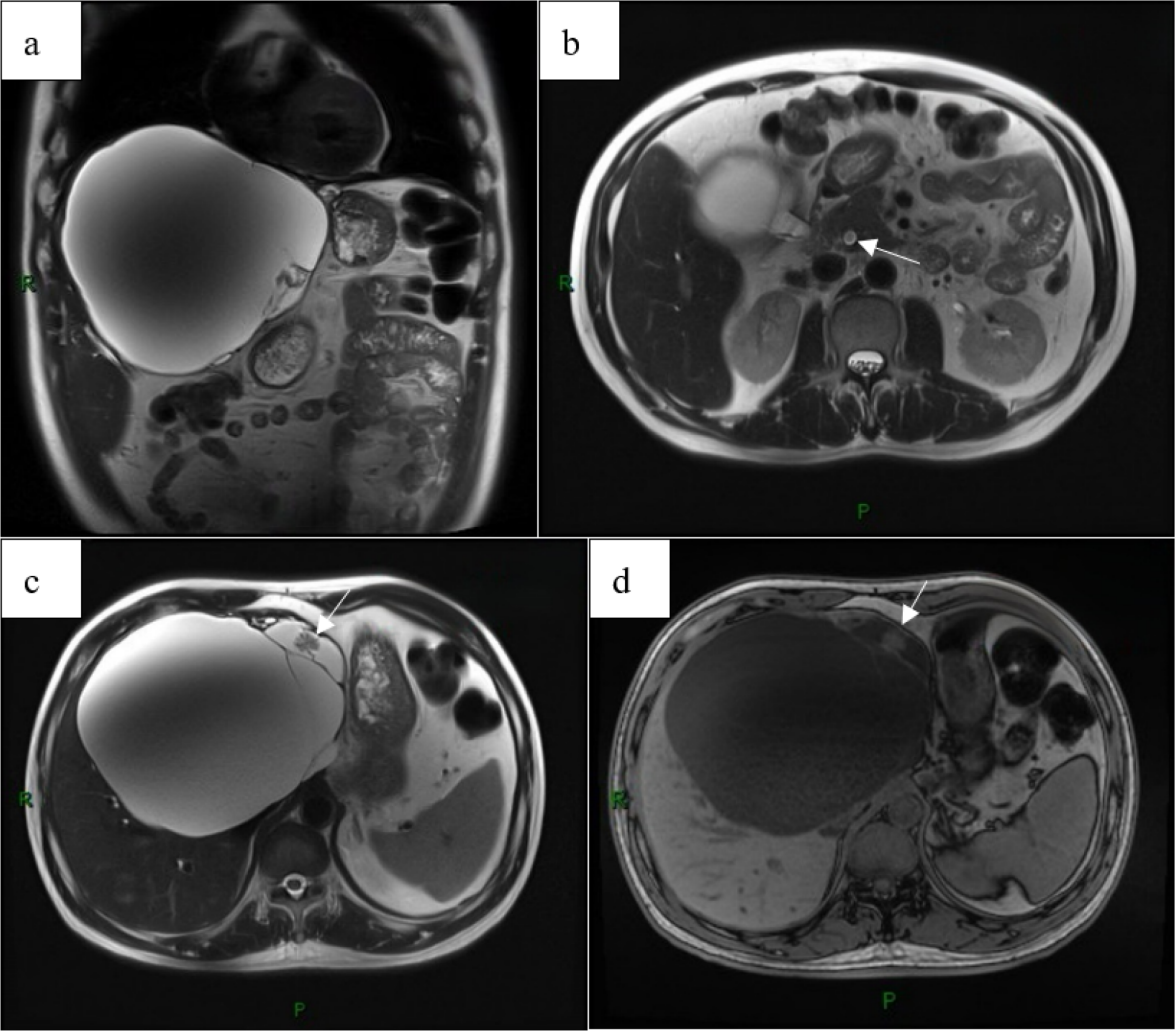
MRI with MRCP demonstrates a large complex cystic mass in the left hepatic lobe measuring approximately 17.3 x 13.9 cm **(a)**, initially suggestive of a giant hepatic cyst. MRCP shows intraluminal filling defects within the common bile duct, consistent with choledocholithiasis **(b)**. Internal septations are present, with a cystic-solid component along the anteromedial aspect of the lesion **(c, d)**, findings suggestive of a mucinous cystic neoplasm.

**Figure 4:**
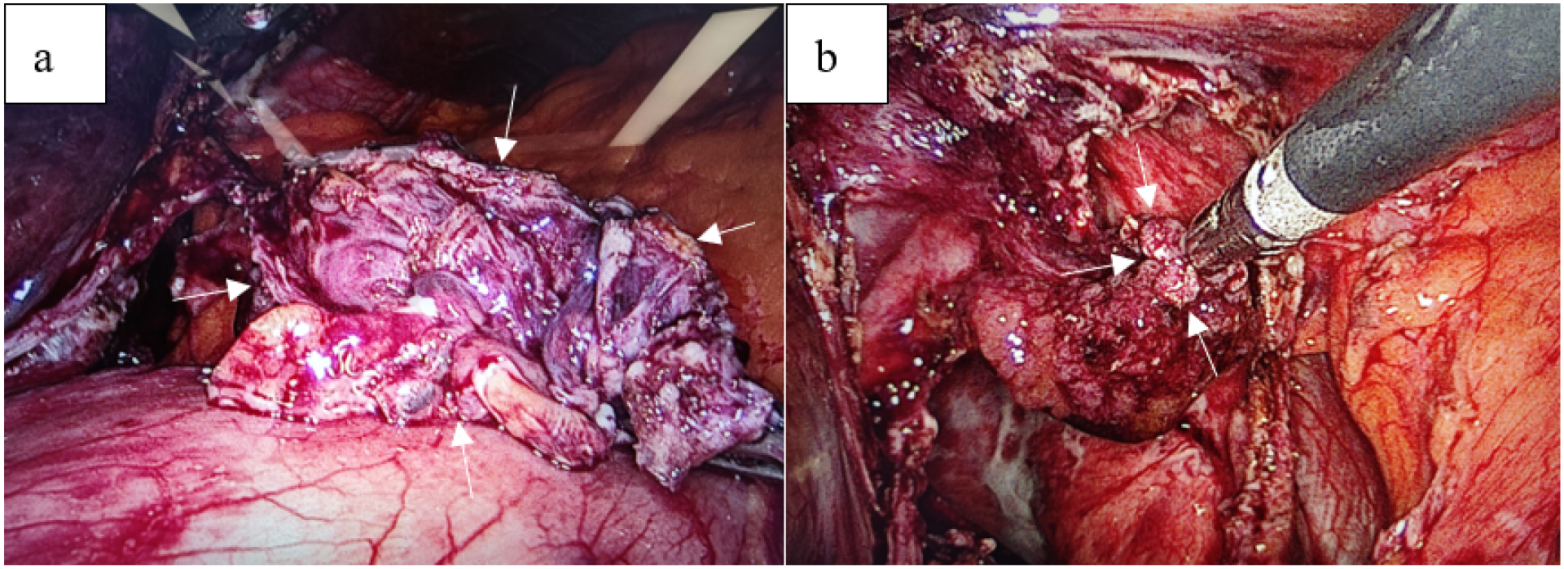
The cyst wall tissue removed during the operation **(a)** and the completely excised tumor tissue **(b)**.

**Figure 5:**
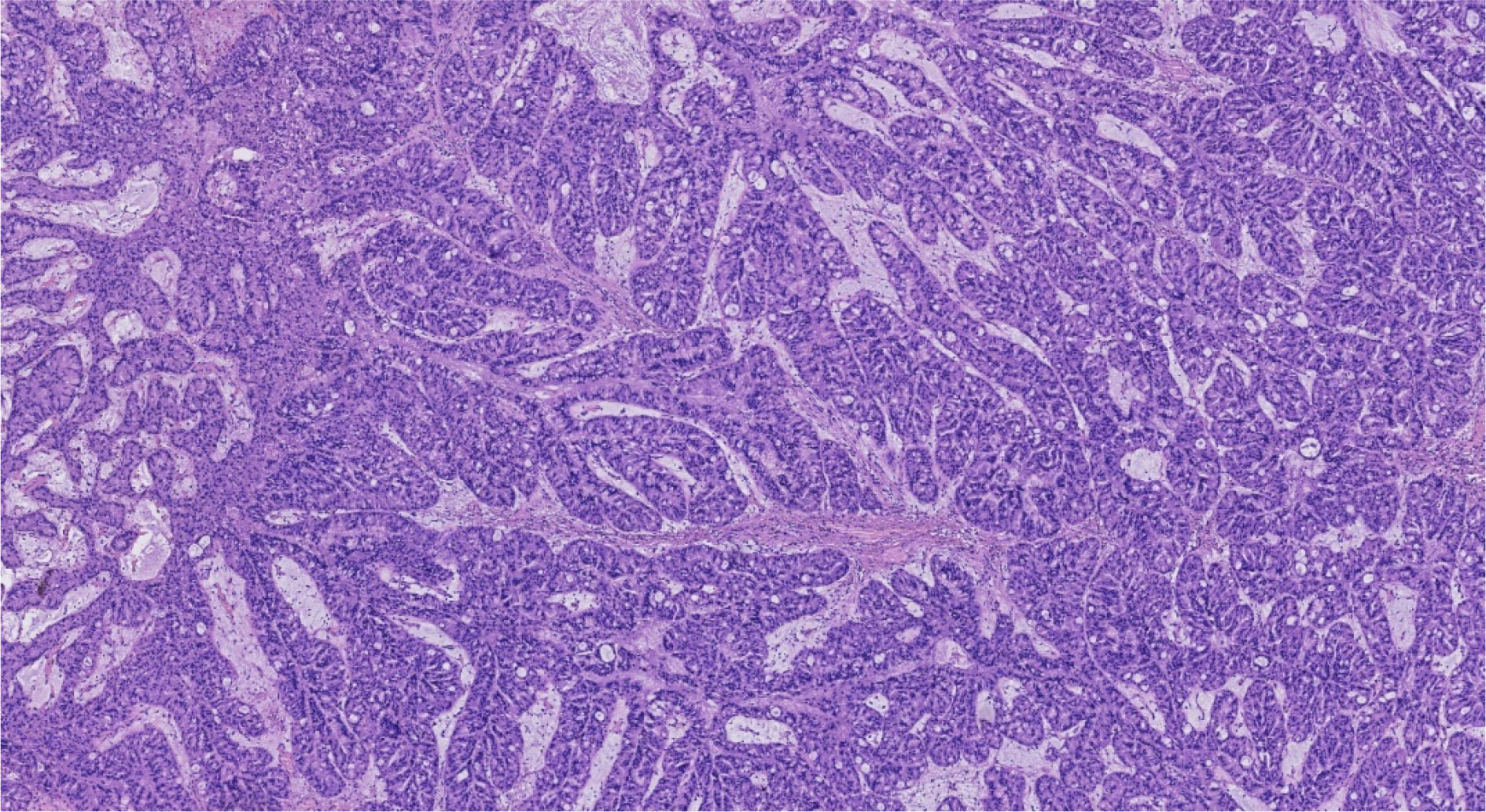
Intraoperative frozen-section analysis demonstrated a tubulovillous adenoma with focal high-grade intraepithelial neoplasia within the glandular epithelium (HEx5).

**Figure 6:**
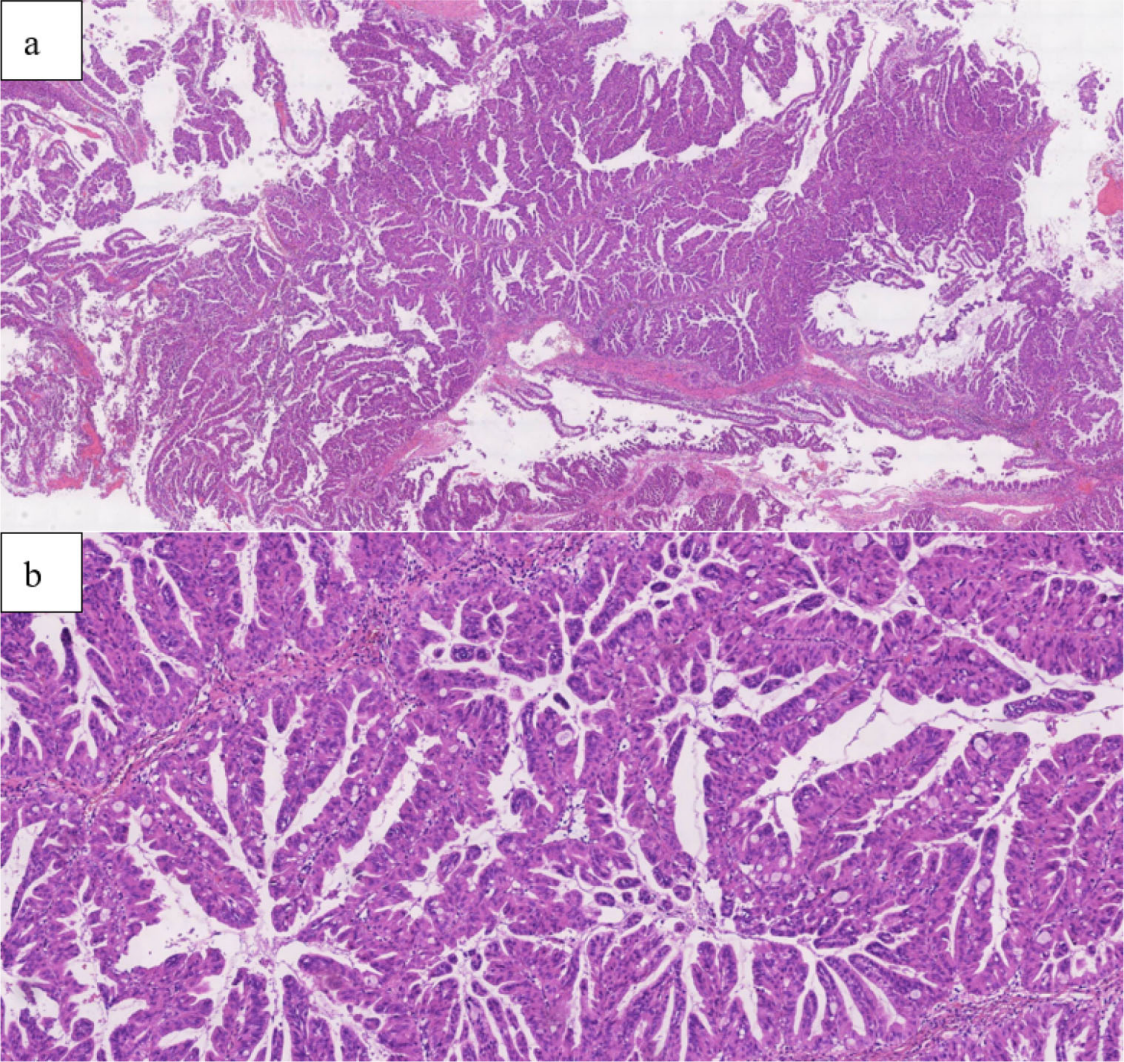
Postoperative pathology (**a**: HEx2, **b**: HEx10) suggested an intraductal papillary tumor with high-grade intraepithelial neoplasia and focal carcinoma (well-differentiated adenocarcinoma), without invasion of blood vessels and nerves.

**Figure 7:**
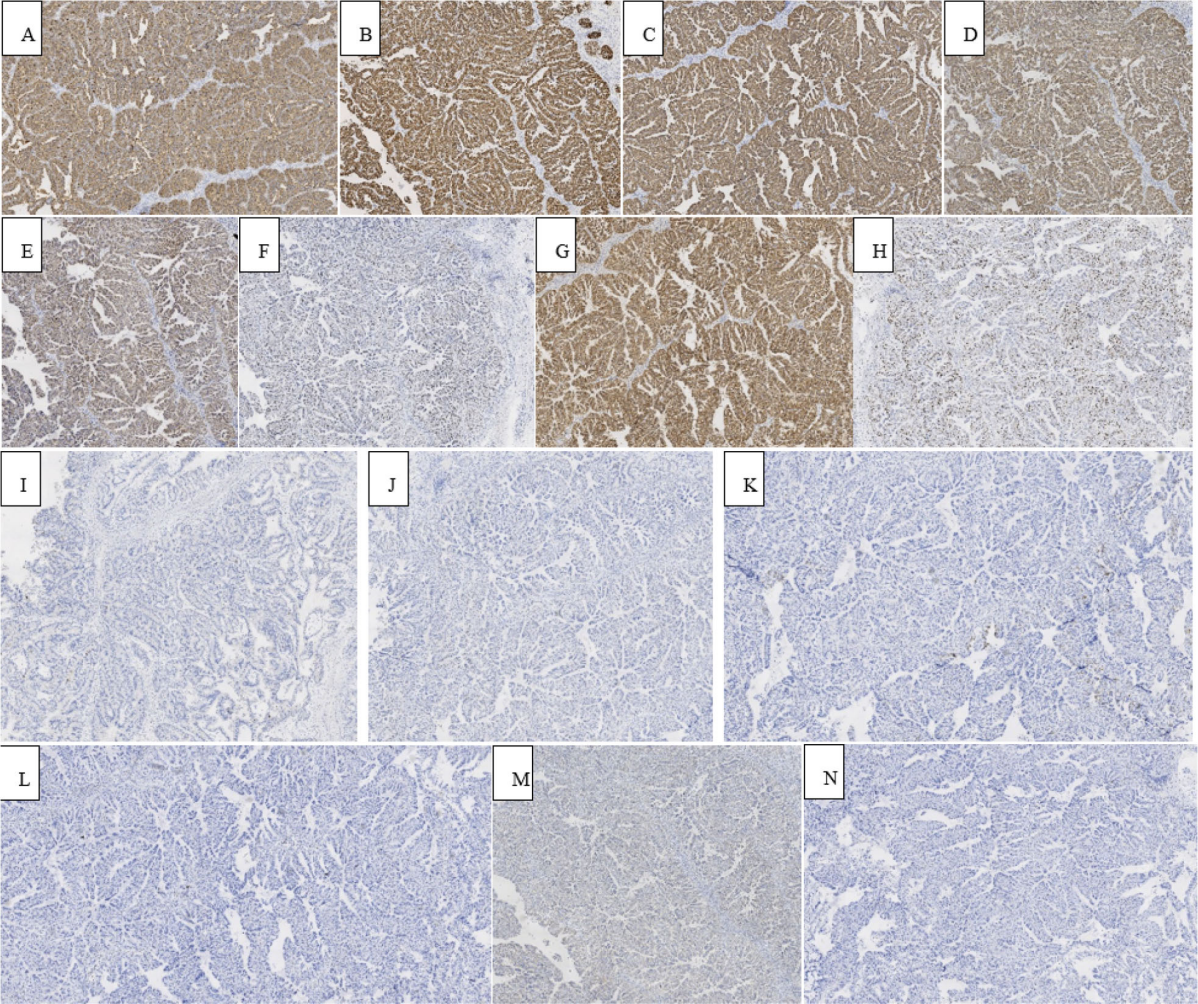
Immunohistochemical staining showed that MUC5AC, MUC6, CK19, CK8/18, HER-2, P53, EGFR, and Ki-67 (15%) were positive in tumor cells **(A–H)**, while MUC2, CDX-2, CK7, CK20, Villin, and CD56 were negative in tumor cells **(I–N)**.

A 54-year-old man underwent CT during a routine health check, which revealed a large hepatic cyst measuring approximately 17 cm in maximal diameter (**Figure 1**). As he was asymptomatic, contrast-enhanced CT of the upper abdomen was recommended for further characterization rather than immediate intervention. He was admitted one month later for diagnostic workup and treatment planning. On admission, the patient had no prior surgical history and no history of infectious or chronic disease. The abdomen was soft, with no palpable masses and no tenderness over the hepatic region. Contrast-enhanced upper abdominal CT demonstrated a round, fluid-attenuation lesion measuring 13.9 × 16.7 cm without internal enhancement (**Figure 2**), consistent with a giant hepatic cyst. Laboratory studies showed liver dysfunction consistent with cholestasis (**Table 1**), likely secondary to biliary obstruction. Upper abdominal MRI demonstrated a 13.9 × 17.3 cm complex cystic–solid mass in the left hepatic lobe with multiple internal septations and T2-hyperintense cystic components, along with intraluminal signal abnormalities within the common bile duct compatible with choledocholithiasis (**Figure 3**). The initial differential diagnosis was a space-occupying lesion—either a giant simple hepatic cyst or a mucinous cystic neoplasm—complicated by common bile duct stones. After excluding contraindications, the patient underwent ERCP with endoscopic sphincterotomy, stone extraction, and biliary stent placement. Subsequently, laparoscopic hepatic cystectomy with partial hepatectomy was performed. The resection included the cyst wall and adjacent liver parenchyma (**Figure 4a**). A discrete area of suspicious tumor was identified (**Figure 4b**) and removed en bloc. Intraoperative frozen-section analysis suggested a tubulovillous adenoma with focal high-grade intraepithelial neoplasia within the glandular epithelium (**Figure 5**). Final histopathology demonstrated intraductal papillary cholangiocarcinoma (IPCC) with high-grade intraepithelial neoplasia and focal well-differentiated invasive adenocarcinoma, without lymphovascular or perineural invasion (**Figure 6**). Immunohistochemical staining demonstrated positive expression of MUC5AC, MUC6, CK19, CK8/18, HER-2, P53, EGFR, and Ki-67 in the tumor cells, while MUC2, CDX-2, CK7, CK20, Villin, and CD56 showed negative results (**Figure 7**). Integrating the imaging, pathologic, and immunohistochemical findings, the final diagnosis was IPCC coexisting with a giant hepatic cyst. A multidisciplinary team recommended extended radical resection with curative intent, followed by systemic therapy (chemotherapy with consideration of targeted therapy or immunotherapy); however, the patient declined further surgery and adjuvant treatment. As of the manuscript revision (5 months post-surgery), the patient remains alive.

**Table 1:** Laboratory studies.

Laboratory study	Result	Reference range
Complete blood test		
Hemoglobin, g/L	151	120--160
Total leukocyte count, × 10^9^/L	5.5	4--10
Neutrophil count	3.3	2--7
Lymphocyte count	1.4	0.8--4.0
Platelet count, × 10^3^/L	120	100--300
Blood urea nitrogen, mg/dL	19.04	8.96--19.88
Serum creatinine, mg/dL	0.88	0.60--1.20
AST, U/L	152	10--40
ALT, U/L	185	10--40
TBIL, mg/dL	1.62	0.29--1.23
DBIL, mg/dL	0.54	0--0.20
Blood albumin, g/L	47.4	35--55
Globulin, g/L	30.4	20--40
Prealbumin, mg/L	194.3	200--400
A/G	1.6	1.5—2.5
Urinalysis		
pH	6.5	4.5—8.0
Specific gravity	1.020	1.003—1.030
Proteinuria	Negative	Negative
Ery	Negative	Negative
U-LEU	Negative	Negative
GLU	Negative	Negative
Nitrite	Negative	Negative
Anti-HIV antibody	Negative	Negative
Syphilis antibody	Negative	Negative
HBs antigen	Negative	Negative
Anti-HCV antibody	Negative	Negative

## Discussion

Cholangiocarcinoma is an aggressive malignancy arising from the biliary epithelium, with a globally rising incidence; it accounts for approximately 15% of primary liver cancers and about 3% of gastrointestinal malignancies (6). Adenocarcinoma accounts for 90–95% of cholangiocarcinomas. By anatomic location, cholangiocarcinoma is classified as ICC or extrahepatic cholangiocarcinoma, the latter further subdivided into perihilar (hilar/Klatskin) and distal cholangiocarcinoma (7). Biliary obstruction can produce cholestatic symptoms, including jaundice, dark urine, acholic (clay-colored) stools, and pruritus. Jaundice is uncommon in ICC unless advanced or central ducts are involved; more typical presentations include right upper quadrant pain, malaise/fatigue, night sweats, unintended weight loss, and cachexia (8, 9).

Hepatic cysts are common benign liver lesions characterized by fluid-filled cavities; most are congenital, and their precise etiology remains uncertain (10). Most hepatic cysts are asymptomatic; however, large cysts may cause dyspepsia, early satiety, anorexia, nausea, and vomiting due to mass effect on adjacent organs or impaired hepatic function (11). During a routine health examination, a previously asymptomatic patient was found to have a giant hepatic cyst. Postoperative histopathological and immunohistochemical analyses of the resected specimen confirmed IPC coexisting with the cyst.

The coexistence of cholangiocarcinoma and hepatic cysts is exceptionally rare and clinically challenging. Giant cysts can compress bile ducts, impairing drainage and causing localized bile stasis that predisposes to secondary bacterial infection. Chronic stasis and infection drive sustained inflammation, epithelial injury, and regenerative hyperplasia of cholangiocytes, fostering dysplasia and malignant transformation and thereby increasing the risk of cholangiocarcinoma (2, 12). Moreover, sustained mechanical compression and irritation of the biliary epithelium can drive repeated cycles of injury and repair, leading to reactive cholangiocyte hyperplasia and dysplasia, thereby promoting neoplastic transformation and tumor development (13). Although high-throughput DNA and RNA sequencing has substantially advanced our understanding of the molecular pathogenesis of cholangiocarcinoma associated with hepatic cysts, these insights have not yet translated into meaningful improvements in patient prognosis.

In 2024, Chang et al. described a 62-year-old man with abdominal discomfort and bloating in whom imaging identified a complex hepatic cyst; liver biopsy demonstrated moderately differentiated adenocarcinoma with tumor necrosis, consistent with ICC (14). Notably, despite biochemical evidence of hepatic dysfunction, serial CA 19–9 levels remained within the normal range. Although CA 19–9 is a conventional biomarker for cholangiocarcinoma (3), its diagnostic performance is limited: levels may be spuriously elevated in benign biliary obstruction/cholangitis and, conversely, normal values do not exclude malignancy, underscoring the need to interpret CA 19–9 in clinical context (2).

In a patient with ICC complicated by a giant hepatic cyst, laboratory tests showed cholestatic liver dysfunction suggestive of biliary obstruction, which was corroborated by MRI. ERCP achieved decompression and stent placement, and the definitive diagnosis was established by postoperative histopathology and immunohistochemistry. These cases underscore the need to integrate laboratory data with high-quality imaging—and proceed to timely endoscopic or surgical intervention with tissue confirmation—to enable early, accurate diagnosis and optimal management.

Ultrasound is the most widely used and accessible first-line imaging modality; it detects hepatic cysts and characterizes hyperechoic or mixed-echoic hepatic lesions (9). CT delineates hepatic anatomy, characterizes lesions, and detects metastatic disease (15). Three-phase CT, including arterial, portal venous, and delayed venous phases, significantly improves diagnostic accuracy (7, 9). Compared with CT, MRI has superior capability for characterizing strictures, masses, and cysts (16). Magnetic resonance cholangiopancreatography (MRCP) clearly visualizes biliary tract abnormalities, which is critical for diagnosing ductal cell carcinoma (17). Therefore, when CT does not provide definitive differentiation in patients with mixed solid-cystic hepatic masses, a comprehensive upper abdominal MRI is recommended.

The patient’s hepatic cystic lesion was detected during a routine physical examination. MRI/MRCP demonstrated a complex cystic-solid mass in the left hepatic lobe accompanied by choledocholithiasis. These findings further underscore the superior diagnostic yield of MRI/MRCP over CT in delineating ductal stenosis, characterizing masses and cystic lesions, and detecting biliary tract pathology. Laboratory studies and imaging were consistent with a benign hepatic cystic lesion and common bile duct stones. Given the patient’s clinical history, abnormal liver function tests were attributed to biliary obstruction from choledocholithiasis. Accordingly, ERCP was performed to relieve common bile duct obstruction prior to definitive surgical management of the hepatic cyst.

Although imaging is pivotal in diagnosis, cholangiocarcinoma presenting with hepatic cysts can closely mimic simple hepatic cysts on imaging. Therefore, definitive diagnosis requires pathologic evaluation. Histopathologic analysis, the gold standard for diagnosing cholangiocarcinoma (18), performed via fine-needle aspiration biopsy or examination of resection specimens, identifies histologic type, grade of differentiation, and lymph node metastasis, thereby guiding subsequent treatment.

Intraoperative frozen-section examination is a commonly used diagnostic tool that informs real-time surgical decision-making. It can help characterize tumors, assess margin status, evaluate lymph node metastasis, and guide the operative approach and extent of resection. Its reported accuracy is approximately 97%–98%, with a discordance rate of about 2.03% compared with final paraffin (permanent) sections (19, 20). Limitations include reduced cytologic detail relative to paraffin sections, inapplicability to certain tissues, and high demands on the pathology team. Discrepancies between frozen and permanent diagnoses—particularly when distinguishing benign from malignant lesions—can directly influence surgical strategy. In this case, although the intraoperative frozen-section diagnosis differed from the postoperative paraffin diagnosis, the margins were negative and the tumor was completely resected, so the discrepancy did not affect the patient’s outcome or the choice of adjuvant therapy.

Surgical resection remains the only potentially curative therapy and is the current first-line approach (3). The primary surgical objective is complete removal of the tumor and involved hepatic parenchyma while preserving adequate healthy liver to maintain function. For patients with unresectable tumors or residual disease after surgery, adjuvant chemotherapy and radiotherapy are crucial. In the BILCAP trial, adjuvant capecitabine for cholangiocarcinoma improved overall survival (21), although this finding requires further statistical validation and additional evidence. Although immunotherapy and molecularly targeted therapy have made significant advances in cholangiocarcinoma, these modalities expand treatment options but still require ongoing refinement (3). Treatment should be individualized to the patient’s clinical status.

Patient responses to a new cancer diagnosis are often conceptualized by the Kübler-Ross stages (denial, anger, bargaining, depression, acceptance) (22), though individual trajectories vary. In this case, the patient appeared to be in an early response phase (denial or anger) and expressed a strong belief that complete (R0) resection had “cured” the disease, leading to refusal of additional evaluation or adjuvant therapy. This decision may also have been influenced by perceived treatment burden and fear of adverse effects. The case underscores the importance of timely psycho-oncologic support, clear communication of prognosis and risk-benefit considerations, and shared decision-making to align care with patient values while reducing the likelihood of treatment refusal.

This report describes a single case with only 5 months of postoperative follow-up. The patient declined additional follow-up and adjuvant therapy, precluding assessment of long-term oncologic outcomes. As a result, the findings have limited generalizability, and longitudinal prognostic data for this rare entity are lacking.

In future clinical practice, early detection and accurate characterization of hepatic space-occupying lesions are crucial. Timely, systematic management can help preserve patients’ hepatic function and overall health and improve prognosis.

## Conclusion

IPC concomitant with a giant hepatic cyst is a rare and complex clinical entity. Diagnosis is challenging; at initial presentation, clinicians may overlook or misclassify the condition, delaying treatment and compromising therapeutic planning. In clinical practice, when imaging reveals hepatic cysts or mixed solid-cystic hepatic masses, clinicians should rigorously assess for malignant hepatic neoplasms and remain vigilant for concurrent pathology.

## Data Availability

The original contributions presented in the study are included in the article/supplementary material. Further inquiries can be directed to the corresponding authors.
